# Erbium Laser for Skin Surgery: A Single-Center Twenty-Five Years’ Experience

**DOI:** 10.3390/medicines8120074

**Published:** 2021-11-24

**Authors:** Steven Paul Nisticò, Giovanni Cannarozzo, Piero Campolmi, Federica Dragoni, Silvia Moretti, Cataldo Patruno, Luigi Bennardo

**Affiliations:** 1 Department of Health Sciences, Magna Graecia University, 88100 Catanzaro, Italy; cataldopatruno@libero.it (C.P.); luigibennardo10@gmail.com (L.B.); 2 Unit of Dermatology, Tor Vergata University, 00133 Rome, Italy; drcannarozzo@gmail.com; 3 Section of Dermatology, Department of Surgery and Translational Medicine, University of Florence, 50120 Florence, Italy; pierocampolmi@gmail.com (P.C.); silvia.moretti@unifi.it (S.M.); 4 Dermatology Unit, San Donato Hospital, 52100 Arezzo, Italy; federicadragoni@yahoo.it; 5 Unit of Dermatology, Mariano Santo Hospital, 87100 Cosenza, Italy

**Keywords:** Erbium laser, Er-Yag laser, seborrheic keratosis, skin scars, facial rejuvenation

## Abstract

(1) Introduction: The Erbium laser is a very versatile laser system used in dermatology. Its ability to be almost selectively absorbed by water makes it a perfect device for managing various cutaneous skin conditions. (2) Methods: In this paper, we report our twenty-five years’ experience with the Erbium laser. More than three thousand patients were treated for common skin disorders such as flat warts, seborrheic keratosis, xanthelasmas, and scars. (3) Results: A complete response was observed in 89.6%, without significant side effects. Local anesthesia was used in only a tiny percentage of patients. (4) Conclusions: This study confirms that the Erbium laser is a valuable and flexible procedure for laser surgery with excellent safety and short healing times.

## 1. Introduction

The usefulness of laser surgery in dermatology is increasingly recognized in treating many skin and mucosal lesions [1]. The Erbium laser was introduced to vaporize and remove tissues and is a versatile laser system used in dermatology [2]. However, its main field of use is dentistry, where this type of laser is a cornerstone in oral surgery and implantology [3,4,5,6]. The Erbium laser emits at 2940 nm and is characterized by an active medium consisting of a Yttrium–Aluminium–Garnet (YAG) crystal doped with erbium ions [7,8]. The 2940 nm wavelength guarantees a maximum absorption by the aqueous tissue component (this wavelength is the closest to the 3000 nm absorption peak of water), thus allowing a very superficial epidermal ablation, a minimal thermal effect, and a limited area of necrosis. At the typical working fluence (around 5–8 J/cm^2^ with average energies of about 300–500 mJ @ 3 mm), the Erbium laser may be used in many superficial interventions [9,10,11,12]. Erbium Yttrium–Aluminium–Garnet (Er:YAG) lasers were first approved in 1996 for cutaneous resurfacing, showing equal efficacy than CO_2_ laser and a more rapid recovery. The pulse length of Er:YAG lasers available on the market is variable, with short-pulsed devices with pulse lengths of 500 microseconds and long pulses devices with pulse lengths as low as ten milliseconds. Er: YAG lasers produce a superficial ablation, affecting mainly the epidermis and papillary dermis. Water vaporization produces a cooling effect, reducing thermal damage to surrounding skin and allowing multiple passages over the same area in the same surgical session. It also decreases the amount of heat and damage to the surrounding tissues. This cooling allows Er:YAG lasers to perform multiple passes over the same skin and ablation area during the same treatment session with minimal heat damage and improved results and recovery. Full ablative therapy or fractional laser skin resurfacing may both be performed with these devices. Different from traditional ablation, fractional treatment generates laser columns in the area while leaving the areas interposed free from treatment. Er:YAG laser resurfacing can be used to manage actinic damage, skin laxity, and dyschromia. In these cases, fully ablative treatment may be proposed to lower Fitzpatrick skin types (I–II), while higher subtypes (III–IV) are usually treated with fractional, more conservative parameters.

Er:YAG lasers have also proven to be very effective in removing benign, premalignant, and malignant skin lesions, using these devices by performing multiple passes over the lesion up to its complete removal. Various conditions have been approached with this method, such as seborrheic keratoses, acrochordons, intradermal cysts, congenital melanocytic nevi in neonates, and acquired melanocytic nevi in adults. Hypopigmentation or hyperpigmentation may result in the treated area [13].

Given a twenty-five-year experience in Erbium laser surgery, we have treated a wide range of patients with numerous dermatological lesions, particularly in delicate sites (periocular and orbit area, perioral site, and external ear). The following paper reports our long-time experience with the Erbium laser compared to data already published in this field.

## 2. Materials and Methods

**Figure 1:**
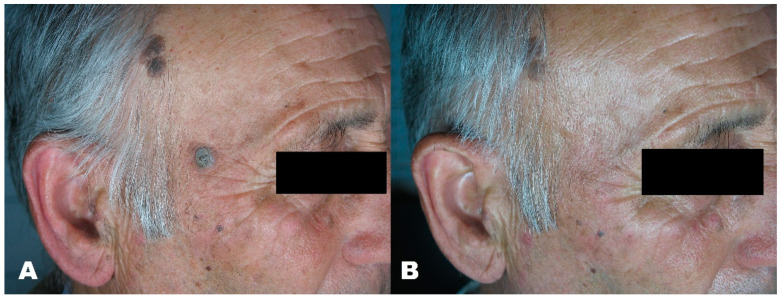
(**A**) Seborrheic keratosis before treatment. (**B**) Region after removal with Erbium laser.

**Figure 2:**
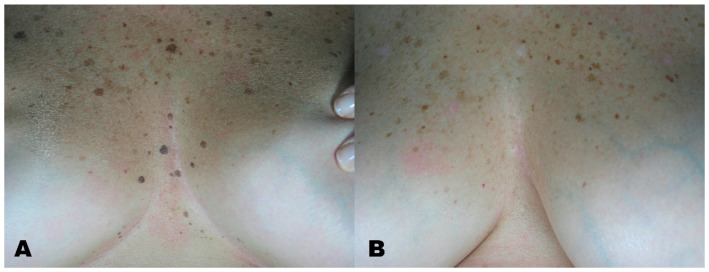
(**A**) Seborrheic keratosis before treatment. (**B**) Region after removal with Erbium laser.

**Figure 3:**
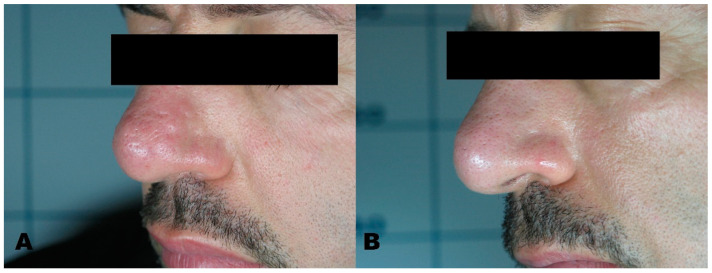
(**A**) Sebaceous hyperplasia before treatment. (**B**) Region after removal with Erbium laser.

**Figure 4:**
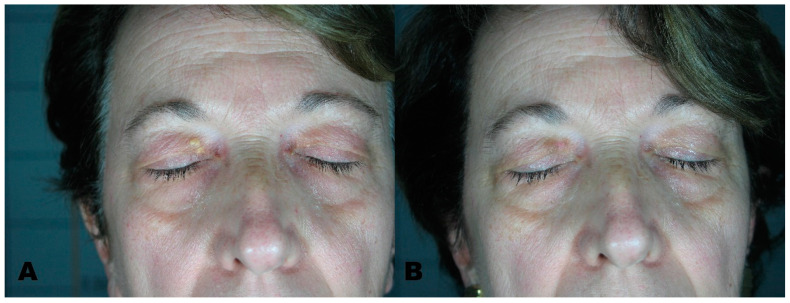
(**A**) Xhantelasmas before treatment. (**B**) Region after removal with Erbium laser.

**Figure 5:**
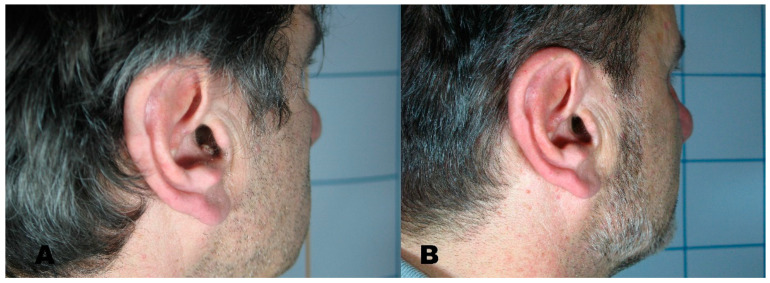
(**A**) Ear wart before treatment, (**B**) Region after removal with Erbium laser.

**Figure 6:**
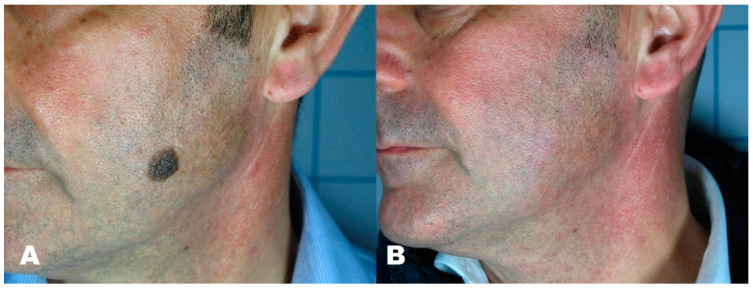
(**A**) Seborrheic keratosis before treatment. (**B**) Region after removal with Erbium laser.

**Figure 7:**
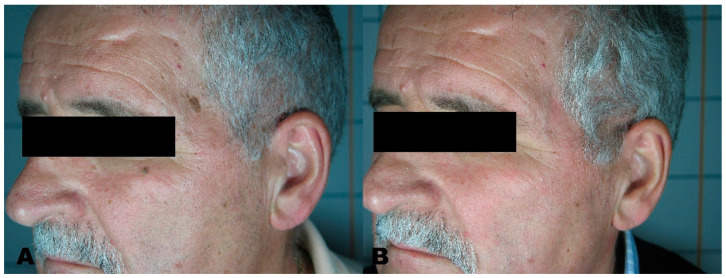
(**A**) Seborrheic keratosis before treatment. (**B**) Region after removal with Erbium laser.

**Figure 8:**
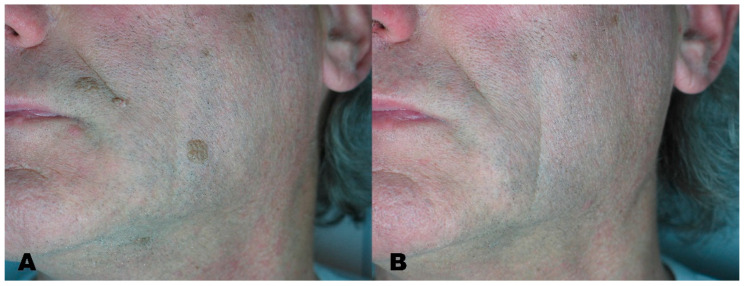
(**A**) Facial flat warts before treatment. (**B**) Region after removal with Erbium laser.

**Figure 9:**
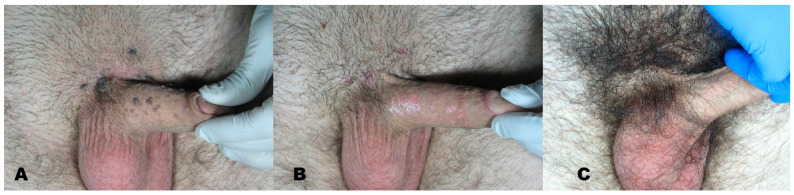
(**A**) Condyloma acuminata before treatment. (**B**) region right after removal with Erbium laser. (**C**) Area at 6-month follow-up.

Over 25 years (1994–2019), about 3123 patients were treated with Erbium laser in our clinic, 1261 males and 1862 females (age range 19–88 years, mean age 46.8 years). Common skin disorders such as flat warts, seborrheic keratosis, xanthelasma, and skin scars were seen and treated (Figure 1, Figure 2, Figure 3, Figure 4, Figure 5, Figure 6, Figure 7, Figure 8 and Figure 9).

After obtaining a detailed personal history (skin type, clinical manifestations, health conditions, previous medications, and lifestyle) and informed consent, patients underwent treatment. Contraindications to treatment include isotretinoin intake in the last six months or other retinoids intake in the last year, radiation therapy, presence of keloid, ectropion, and the presence of cutaneous conditions that could potentially trigger a Koebner phenomenon, such as vitiligo.

All lesions were treated with two 2940 nm Erbium laser systems (Smart 2940+ and Erise of Luxea platform, DEKA-M.E.L.A., Calenzano, Italy) with an operational fluence of 1–8 J/cm^2^ and 1–10 Hz frequency, spot size 2–9 mm depending on the location, type, and level of lesions. In the case of use of the laser for facial acne scars and skin resurfacing, to avoid the risk of herpes reactivation, acyclovir 400 mg cpr (Zovirax^®^, GlaxoSmithKline S.p.A., Milan, Italy) was prescribed three times a day per 10 days, starting 48 h before treatment. Pain during the procedures was acceptable, as most lesions were treated without anesthesia. An effective cooling device was constantly used during each laser session, improving comfort.

To treat the most delicate areas, a lidocaine 2.5%, and prilocaine 2.5% anesthetic ointment was applied under occlusion two hours before treatment (thirty minutes for mucosal lesions) and removed before the procedure. If pain was present even after topical anesthesia, local anesthesia with 2% lidocaine was performed. The patients were asked for allergic reactions to lidocaine and prilocaine prior to topical/local anesthesia. After treatment, topical fusidic acid (Fucidin^®^, Leo Pharma S.p.A., Roma Italy) was used and was applied in the treated area at home twice a day for seven days to avoid potential superinfections. Patients had to avoid sun and cosmetics during the immediate post-procedural periods and apply sunscreens until complete recovery when photo exposed areas were involved.

Data analysis (mean, standard deviations, and rate calculations) was performed using Statistica 14.0 (TIBCO Software, Palo Alto, CA, USA). Patients were followed up for an average period of six months to document possible relapses and adverse events.

## 3. Results

After laser treatment, a complete response was observed in 89.6% of patients, with almost all patients observing global improvement. In our experience, immediate side effects consist of pain, slight erythema, and edema, but most patients (2780 out of 3123, 89%) tolerated the treatment well. Moreover, bleeding and herpes virus reactivation may present as short-term complications, although a cycle of antiviral therapy may reduce the risk of viral reactivation. The whole epithelialization period was in a range of 5–9 days, and the mean duration of erythema was 7–10 days.

No wound infection was observed. Post-inflammatory hyperpigmentation and hypopigmentation were seen in 125 (4%) and 62 (2%) of patients, respectively, probably due to poor sunscreen application in phototype four subjects. Four patients (2.58%) developed retracting scars of the inner part of the upper eyelid after the treatment of xanthelasmas.

Six months’ follow-up showed a recurrence in 6% of cases (187 patients). Eighteen patients with warts showed a relapse within three months after treatment (0.57%).

Esophitic lesions such as sebaceous adenomas, seborrheic keratosis, neurofibromas, warts, condylomas, and trichoepithelioma were treated with the following parameters: 1–5 J/cm^2^ with a 2–9 mm spot to ablate the lesion, causing the possible minor damage to surrounding tissues. Acne scars and skin resurfacing were treated using fractional mode with the following parameters: 0.4–0.6 J/cm^2^ with a 9 mm spot (fractional mode). Harder to treat lesions, such as rhinophyma, xanthelasma, or actinic cheilitis, were treated by attempting to convey energy to the areas with particular attention in order to reduce the risk of scarring in these areas, which may result in functional problems (retracting scars, erosions, etc.); the parameters used were: 1–3.5 J/cm^2^ with a 2–4 mm spot. Satisfactory results were obtained in almost 90% of patients, even in lesions present in challenging areas.

Patient characteristics and satisfaction are reported in Table 1.

## 4. Discussion

This paper reports our twenty-five years’ experience in using an Erbium laser to treat many superficial skin pathologies. The study has evidenced an overall patient improvement that confirms the Erbium laser as a valid procedure for laser surgery.

Physical processes regulate the interaction between the electromagnetic radiation emitted by a laser source and biological tissues, the exchange of energy between the wave and the substrate, and the targeted tissue’s biological response [14].

Water is the skin’s main component (about 77% of its volume), so it plays a fundamental role in laser-tissue interaction. Surgical lasers emit in the infrared spectrum region where, compared to penetration, the absorption of radiation by the water molecules prevails [15,16]. The primary objective of a surgical laser is to reach a target with minimal thermal damage. This goal is usually achieved by vaporizing the tissue in less time than it takes for heat to diffuse (i.e., in a shorter thermal relaxation time (TRT). This consideration is crucial for the correct use of surgical lasers: pulses with high power and very short duration generate minor thermal damage [14].

The Erbium laser works emitting a 2940 nm wavelength and has a very high water absorption (16 times more than the 10,600 nm wavelength of a CO_2_ laser) [17,18]. Therefore, the penetration depth of the Erbium laser is minimal, confined to the uppermost parts of the epidermis, associated with a minimal thermal injury in the adjacent tissues [19].

Acne scars and facial rejuvenation are two hot topics in dermatology; various lasers are currently used to manage these cosmetic issues, ranging from devices selectively targeting collagen [20,21] to intense pulsed lights and fractional lasers [22].

Fractional lasers produce thermal columns that induce heating and denaturation of collagen with subsequent neo-collagen genesis (biostimulation) in the treated areas [23]

These features make the Erbium laser suitable for many skin surface interventions, from resurfacing treatments to the vaporization of numerous benign dermatological lesions in sensitive areas [24,25].

The Erbium laser’s reduced depth of ablation implies the need to carry out multiple laser passes. Therefore, the clinical end-point is no longer based on the feedback of the color indicators (as in CO_2_) but on evaluating non-objective visual parameters related to operator experience [26].

These two lasers may also be used in a combined mode, with a decreased incidence of crusting and pruritus following treatment compared with treatment with CO_2_ lasers alone [27].

In the literature, many studies have reported the effectiveness of the Erbium laser in resurfacing [28] and in vaporizing hyperkeratotic lesions, [29] stressing its differences from CO_2_ laser surgery [30,31,32].

Khatri treated over 360 lesions in 27 patients using this device. Among the lesions treated, the following were also present: acrochordons, nevi, milia, xanthelasmas, seborrheic and actinic keratosis, solar lentigos, verrucae, syringomas, and sebaceous nevi. Moreover, a Bowen disease was treated, showing no sign of relapse. Only eight lesions (2.2%) relapsed, five sebaceous hyperplasias, and three solar lentigos. Laser removal of malignant or premalignant lesions should be performed with great care and limited to superficial basal cell carcinomas or in situ squamous cell carcinomas if the patient refuses surgical excision. The laser removal of nevi should also only be performed in cases where the lesion can be surely classified as benign [33].

One of the first proposed dermatological indications of Er:YAG laser was the management of actinic keratoses. The first study recruited five patients with multiple facial actinic keratoses treated using two to three passes of the laser. All patients showed a reduction in precancerous lesions ranging from 86% to 89%, with re-epithelialization occurring after 5–8 days and erythema lasting up to 6 weeks after the procedure. A histologically proved reduction in solar elastosis three months after treatment was also noted [34].

Ostertag et al. used Er:YAG laser to treat congenital melanocytic nevi in newborns. Ten infants affected by a congenital epidermal nevus were recruited immediately after birth. Six patients presented the lesions on the scalp, while the other two patients presented the lesions on the trunk and the remaining newborns on the extremities. Treatments were performed mainly in general anesthesia, in a single session when the nevi interested the extremities, and in multiple sessions (due to the significant extension of the lesions) when they involved the trunk. The researchers experienced good results, with no or minimal repigmentation, in eight of ten patients and with very few side effects such as postoperative pain, bleeding, and scarring. The removal of congenital melanocytic nevi with laser remains a controversial topic due to the potential risk of malignant transformation of this kind of lesion [35].

Ablative skin lasers are usually used with caution in patients with higher Fitzpatrick skin phototypes (five and six) due to increased risk of changes in pigmentations. Er:YAG laser seems to be more effective in reducing this risk. Davis reported the treatment of milia in black skin using a short-pulsed Er.Yag laser with no changes in pigmentation, suggesting this kind of device as a viable alternative for patients with darker skin. This result was probably due to reducing the inflammatory phase compared to CO_2_ and long-pulsed Er:Yag lasers [36].

An Iranian study compared using cryotherapy, electrodesiccation, CO_2_ laser, and Er:YAG laser in the management of seborrheic keratoses. Thirty patients affected by facial seborrheic keratoses were randomly allocated in one of four groups treating the lesions with different methods, with therapeutic results evaluated eight weeks after surgery through clinical and dermatoscopic exams. The improvement rate was significantly higher in the CO_2_, Er:YAG lasers and electrodesiccation group compared to the cryotherapy at the dermatological follow-up. However, no significant difference emerged among these three groups. The patients reported statistically significant lower satisfaction rates in the cryotherapy group compared to all other treatments. The authors also observed no difference in post-inflammatory pigmentation changes among groups and reported an unexpected longer duration of erythema in the Er:YAG group [37].

An Austrian study compared the use of electrodesiccation, CO_2_ laser, and Er:YAG to remove multiple cutaneous neurofibromas in von Recklinghausen’s disease. In this perspective paper, 15,580 neurofibromas were removed via electrosurgery, CO_2_-, or Er:YAG laser ablation in 21 patients. In adjacent test areas, the researchers compared thermal necrosis, postoperative pain, re-epithelialization, length of postoperative erythema, and the cosmetic outcome of the treatments. Re-epithelization was shorter for Er:YAG laser (9.8 days) compared to CO_2_ laser (20.8 days) and diathermy (27 days). Post-treatment erythema was shorter after Er:YAG laser treatment (8.2 weeks) compared to CO_2_ laser (11.9 weeks) and diathermy (17 weeks), with the latter method constantly producing hypopigmented scars, while Er:YAG laser seemed the best way to prevent any kind of scars and guaranteed an optimal aesthetic outcome. The recurrence rate of the lesions was low with all methods, and patients were altogether satisfied with the results, with only one patient lamenting poor results after surgery [38].

Various American research groups used fractional Er:YAG laser to manage hypertrophic burn scars and post-traumatic scars. The results of this laser treatment seem promising, with a reduction in erythema and re-epithelization rate. A significant improvement was noted in all measured parameters, including dyschromia, atrophy hypertrophy, vascularity, and texture [39,40].

Fractional Er:YAG laser was also proposed to manage acne scars, with 25 patients receiving four treatment sessions at a one-month interval. Result assessment was performed using the Goodman and Baron grading. Ninety-six percent of patients showed good to an optimal outcome, with patient satisfaction higher than physician’s photographic evaluation [41].

A split-face prospective trial compared the effectiveness of fractional CO_2_ laser and Er:YAG laser to treat acne scars. Twenty-four subjects with acne scars were treated with a fractional Er:YAG laser on one side and a fractional CO(2) laser on the other side of the face. Two treatments with a 2-month interval were performed for every patient. The results assessment was performed one, three, and six months after the last procedure. Scars’ improvement progressed significantly from 1- to 6-month follow-up. At the six-month follow-up, 55% of Er:YAG treated patients and 65% of CO_2_ treated patients reported a 50% or higher improvement in scar presentation, although this difference was not statistically significant. Greater discomfort, however, was experienced by the patients treated with the 10,600 nm wavelength device [42].

The Er:YAG laser was proposed as a treatment for actinic cheilitis. An Israeli group treated twelve patients affected by this condition using Er:YAG laser, experiencing the resolution of the condition in all patients, with no recurrence of the disease in a mean 24 months follow-up, no postoperative complications, and a mean healing time of 22 days [43]. Another group treated an even bigger cohort of patients (99) and, using telephone interviews, followed them up to assess the recurrence rate over time. The mean follow-up time of patients was 66 months, and in this period, 85% of them remained disease free. 92.2% of the respondents believed there had been an improvement in the cosmetic appearance of their lips, and 93.5% were satisfied with the results of laser treatment. Scarring following laser therapy appeared in 5% of patients, showing how it may be challenging to treat this condition in the perioral area [44].

Compared to the CO_2_ laser, which generates more significant thermal damage, Erbium laser intervention is less painful and complications such as delayed re-epithelialization, persistent erythema, dyschromic, and scarring outcomes are less frequent [38]. The advantage of the occasional use of anesthetics makes this laser a great alternative in patients with several lesions and patients who cannot undergo local anesthesia [45]. The major limitation to using the Erbium laser is its minimal coagulation capacity, which often brings difficulties over large areas or easily bleeding neoformations [30,31,38].

This paper has confirmed the efficacy and versatility of the Erbium laser, which results in low postoperative morbidity (hyperpigmentation 4%, hypopigmentation 2%, retracting scars 0.12%), and low risk of complications, and a short recovery period (5–9 days). Excellent results are achievable with this laser: up to 89.6% of patients observed a complete clinical response, and all patients observed global improvements. In a 4-months follow-up, complete remission in 86% of patients was confirmed, and the recurrence of viral warts (0.57%) was not more frequent than other techniques [46].

## 5. Conclusions

Our findings confirm that the Erbium laser may be considered a practical and flexible tool for the laser surgeon in treating many superficial dermatological conditions. Its excellent absorption and low thermal injury can offer a precise ablation with excellent safety and shorter healing times. Due to a longer learning curve than the CO_2_ laser, however, Erbium laser results may be operator-dependent, and in cases of multiple passes, the risk of scarring is still present. Future multicentric studies will be necessary to assess further the results we showed in this study.

## Figures and Tables

**Table 1:** Demographic data of included patients.

Skin Diseases	Number of Lesions Treated	Females	Males	Patients Reporting Complete Response/High Aesthetic Satisfaction	Percentage of Patients Satisfied
Sebaceous adenoma	131	55	76	123	93.9%
Seborrheic keratosis	735	466	269	726	98.8%
Actinic keratosis	234	67	167	186	79.5%
Actinic cheilitis	36	16	20	16	44.4%
Acne scars	196	157	39	167	85.2%
Skin Resurfacing	409	338	71	396	96.8%
Chondrodermatitis nodularis helicis	59	31	28	38	64.4%
Superficial Dyschromias	312	204	108	306	98%
Favre-Racouchot’s disease	39	3	36	18	46.2%
Neurofibroma	98	19	79	77	78.6%
Dermal melanocytic lesions	34	14	20	23	67.6%
Epidermal melanocytic lesions	96	67	29	81	84.4%
Glandular rhinophyma	83	9	74	57	68.7%
Syringoma	71	45	26	63	88.7%
Trichoepithelioma	27	22	5	18	66.7%
Condiloma	121	30	91	107	88.4%
Flat wart	133	96	37	122	91.7%
Filiform wart	76	54	22	69	90.8%
Plantar wart	78	67	11	74	94.9%
Xanthelasma	155	102	53	132	85.2%
Total	3123	1862	1261	2799	89.6%

## Data Availability

Data are available from the corresponding author upon reasonable request.
